# In situ structures of rotavirus polymerase in action and mechanism of mRNA transcription and release

**DOI:** 10.1038/s41467-019-10236-7

**Published:** 2019-05-17

**Authors:** Ke Ding, Cristina C. Celma, Xing Zhang, Thomas Chang, Wesley Shen, Ivo Atanasov, Polly Roy, Z. Hong Zhou

**Affiliations:** 1 Department of Bioengineering, University of California, Los Angeles, CA 90095 USA; 2 California NanoSystems Institute, University of California, Los Angeles, CA 90095 USA; 3 Department of Microbiology, Immunology and Molecular Genetics, University of California, Los Angeles, CA 90095 USA; 4 Department of Pathogen Molecular Biology, London School of Hygiene and Tropical Medicine, London, WC1E 7HT UK

**Keywords:** Transferases, Transcription factors, RNA, Cryoelectron microscopy, Rotavirus

## Abstract

Transcribing and replicating a double-stranded genome require protein modules to unwind, transcribe/replicate nucleic acid substrates, and release products. Here we present in situ cryo-electron microscopy structures of rotavirus dsRNA-dependent RNA polymerase (RdRp) in two states pertaining to transcription. In addition to the previously discovered universal “hand-shaped” polymerase core domain shared by DNA polymerases and telomerases, our results show the function of N- and C-terminal domains of RdRp: the former opens the genome duplex to isolate the template strand; the latter splits the emerging template-transcript hybrid, guides genome reannealing to form a transcription bubble, and opens a capsid shell protein (CSP) to release the transcript. These two “helicase” domains also extensively interact with CSP, which has a switchable N-terminal helix that, like cellular transcriptional factors, either inhibits or promotes RdRp activity. The in situ structures of RdRp, CSP, and RNA in action inform mechanisms of not only transcription, but also replication.

## Abstract (web summary)

Rotaviruses are of great medical significance because they cause gastroenteritis in children. Here the authors provide insights into the mechanism of viral mRNA transcription by determining the in situ cryo-EM structures of a working rotavirus’ RNA-dependent-RNA polymerase, which is of interest for antiviral drug design.

## Introduction

DNA replication and RNA transcription are two of the three steps of Crick’s central dogma governing cellular life^1^. The gradual emergence of DNA-based life forms from the RNA world has been hypothesized to be punctuated by major leaps, including RNA replication, RNA-dependent RNA transcription, and RNA reverse transcription to synthesize DNA^2^. Although ribozymes are rare in the modern world, recent discoveries^3^ have supported the theory that the first RNA-dependent RNA polymerase (RdRp) was likely a ribozyme^4–6^. In the modern DNA-protein world, proteins have evolved to be the preferred polymerases that catalyze DNA replication and RNA transcription, including RNA-dependent RNA transcription occurring in viruses and cells. The first atomic structure of a polymerase (*Escherichia coli* Polymerase I) revealed a characteristic core shaped like a right hand^7^. Crystal structures of viral RdRps^8,9^, such as those in poliovirus^10^, bacteriophage phi6^11^, animal reovirus^12^, and rotavirus^13^, also have cores similar to that of DNA polymerases. A similar core structure also exists in telomerase reverse transcriptase (TERT)^14^. The conserved function of the core is to take a single-stranded nucleotide template and amplify it to a double-stranded product. These polymerases are specialized by both the addition of peripheral domains surrounding the core and the binding of regulatory factors at different time points of polymerization. In the spatial dimension, polymerases that carry out DNA replication (such as DNA polymerase III) contain an exonuclease as a peripheral domain to proofread the dsDNA product; those involved in RNA transcription (such as the viral RdRp of influenza B) possess endonuclease and cap-binding peripheral domains to direct the primer into the active site^15^. In the temporal dimension, this specialization can be further reflected by various regulatory factors, which form various complexes with the polymerase at different stages of polymerization. For example, the RdRp of bacteriophage Qβ recruits host translation elongation factors to form replicase holoenzyme^16^.

In order to fully understand these specialization processes, detailed in situ structures of polymerases in its active states are needed. However, there have been issues with obtaining the correct spatial and temporal contexts for these structures. Reoviruses have long served as model organisms for studying viral RdRp and RNA conservative transcription. Structures of Reovirus RdRp with various RNA substrates^12,13^ have been resolved previously by X-ray crystallography, all of which have a cage-like structure with a cap-binding site and four channels: template entry, NTP entry, template exit, and transcript exit. However, many purified RdRp only shows binding affinity to RNA/NTP substrates and limited polymerization activity^17^, leaving the spatial context unknown. Additionally, previous studies^18,19^ on active reovirus polymerases also failed to show the complete trajectory of the template or transcript RNA, thus leaving unclear the function of potential RNA-interacting peripheral domains (i.e., N- and C-terminal domains in reovirus RdRp). Previous research into these structures has also left unclear the temporal context of these polymerases that undergo conservative transcription (in which the nascent strand is the transcript). Some dsRNA viruses that conduct conservative transcription cannot achieve full polymerase activity by itself. For example, the inner capsid shell protein (CSP) is required for rotavirus’ RdRp to be active in vitro^20^. On the other hand, for some dsRNA viruses that conduct semi-conservative transcription, in which the nascent strand is part of the dsRNA genome (e.g., bacteriophage φ6^21^ and picobirnavirus^22^), RdRp is completely functional for replication in vitro. However, exactly how CSP regulates^23^ RdRp’s activities in rotaviruses remains unknown. Also, unlike other RdRps that conduct semi-conservative transcription, reovirus’s RdRp can conduct both replication and transcription and switch between the two states directly after polymerization. In essence, a virus must be actively running to understand the temporal context, which is very difficult to do through X-ray crystallography.

Cryo electron microscopy (cryoEM) offers opportunities to address both these issues, as it enables the structural characterizations of in situ structures in transient, active states. Here, we report the in situ near-atomic resolution structures of RdRp before and during transcription in rotavirus double layered particles (DLP). Compared to other viruses in the *Reoviridae* family, rotaviruses are of particular interest for several reasons. In terms of medical significance, they cause diarrhea responsible for up to half a million children deaths annually^24^. Rotaviruses also display significant biochemical simplicity, as their RdRp does not have a separate NTPase protein bound as in other reoviruses; thus, the working mechanisms of rotavirus’s RdRp can be studied clearly.

## Results

### In situ structures of RdRp in action

**Fig. 1:**
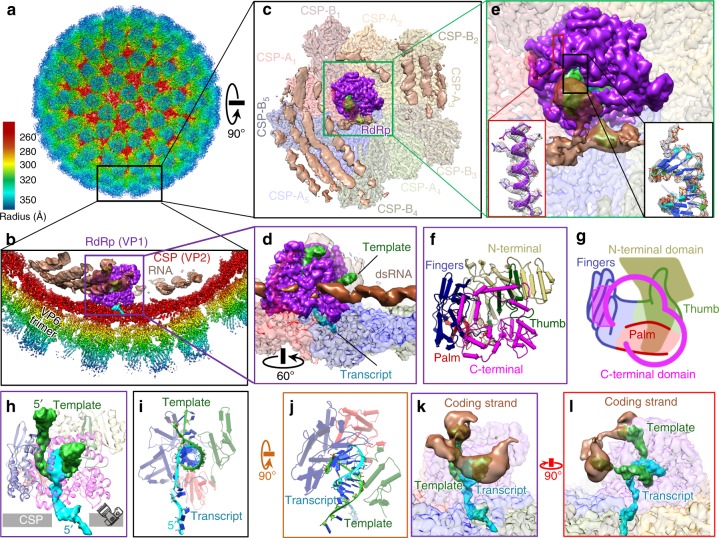
Visualizing a working polymerase in situ. **a** CryoEM reconstruction of rotavirus at 3.4 Å resolution, colored by radius. **b** The RdRp (purple) can be found on the inside of the penton formed by the capsid shell protein (CSP) (red). Genomic RNA density (brown) is packed in the interior of the DLP. The transcript (cyan) is released through the vertex. **c** 90° rotated view from the boxed region in (**a**), showing a classic top view with the extended dsRNA genome (with the typical ~27 Å distance between its neighboring strands). **d** 60° rotated view from the boxed region in (**b**), showing the clear major and minor grooves of dsRNA. **e** Magnified view from the boxed region in (**c**), with additional zoomed-in boxes showing densities (meshes) superimposed upon the atomic models for RNA and RdRp. **f**, **g** Pipes-and-planks representation (**f**) and schematic (**g**) of RdRp in the same classic front view in (**b**), colored by domain. **h** Ribbon models of RdRp during transcription with RdRp shown as ribbons and RNA densities as colored surfaces, including the template (lime green) and transcript (cyan). **i** View from the camera angle shown in (**h**) along the dsRNA axis, shown along with the pipes-and-planks representation for RdRp’s core. **j** 90° rotated view from (**i**) showing the 10-base pair-long dsRNA product. **k** A transcription bubble is formed by template RNA (green) and genomic RNA (brown). **l** 90° rotated view from (**k**) showing the transcription bubble in near proximity to RdRp

To capture RNA transcription in action, we imaged DLPs of rhesus rotavirus (RRV) under active transcribing conditions (Supplementary Fig. 1 and Supplementary Table 1). We resolved RdRp and RNA structures following a two-step data analysis procedure (Supplementary Fig. 2). First, conventional icosahedral refinement of these particles provided a reconstruction at 3.4 Å resolution. To resolve the RdRp, we carried out localized reconstructions^25^. The final localized reconstruction from sub-particles reached 3.6 Å resolution, which showed RdRp (VP1) interacting with both RNA and inner capsid proteins (VP2) (Fig. 1a–d). An atomic model was built based on this high-resolution in situ structure, with distinct side chain densities and RNA features (Fig. 1e, Supplementary Figs. 3 and 4, and Supplementary Movies 1 and 2). We determined that the RdRp is attached to CSP decamers at a specific, off-centered location, as previously described^26,27^. For the ten CSPs in the decamer, we named the five copies close to the decamer center CSP-A_1–5_, and the others CSP-B_1–5_, with respect to its relative position to the RdRp (Fig. 1c and Supplementary Fig. 3). The RdRp has a conserved hand-shaped core domain (residues 333–778), which is sandwiched between an N-terminal domain (residues 1–332) and a C-terminal domain (residues 779–1088) (Fig. 1f, g and Supplementary Movie 3). This core domain can further be divided into the fingers, palm, and thumb subdomains, with the active site located between the fingers and palm. Based on the double-stranded RNA (dsRNA) product density in the active site, we identified two partially-paired single-stranded RNA (ssRNA) strands: the (+)RNA transcript (cyan) and the (−)RNA (lime green) template (Fig. 1h–j). The 5′ end of the transcript extends outside the RdRp, passing through the capsid shell towards the exterior. In contrast, the template strand traverses through the RdRp (parallel to the capsid shell) and reanneals with its complementary coding strand [(+)RNA, brown] to complete a transcriptional bubble within the capsid interior (Fig. 1k, l). Based on these observations, we conclude that our transcribing DLPs are in a transcript-elongated state (TES) and rotavirus is indeed conducting a conservative transcription.

**Fig. 2:**
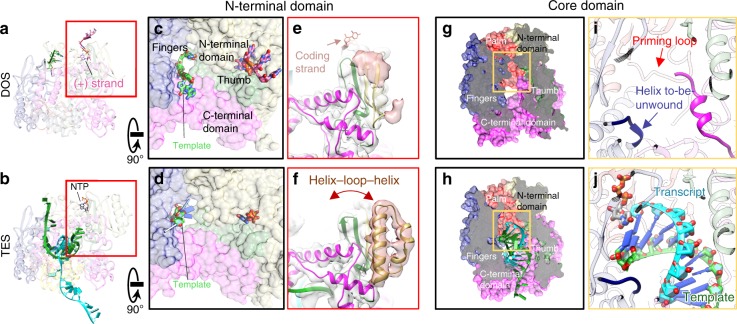
RNA and RdRp conformational changes between DOS and TES. **a**, **b** Ribbon models RpRp (pale) and RNA/NTP (bright) of DOS (**a**) and TES (**b**) from the classic front view. **c**, **d** 90° rotation from (**a**) and (**b**) showing the cap-binding site. The m7G(5′)ppp(5′)GGC cap (hot pink) binds to the N-terminal cap-binding site in DOS (**c**), and is replaced by an NTP (black) in TES (**d**). **e**, **f** Different conformations of the helix-loop-helix subdomain within the RdRp’s N-terminal domain in DOS (**e**) and TES (**f**). **g**, **h** Clipped view of (**c**) and (**d**) showing the active site in DOS (**g**) and TES (**h**). The active site contains no RNA in DOS, but is occupied by both the dsRNA product and incoming NTP in TES. **i**, **j** Magnified view from the boxed regions in (**g**) and (**h**) shows that the active site is partially blocked by the C-terminal domain in DOS, with the priming loop (residues 489–499) retracted (**i**); in TES (**j**), the active site contains the elongated transcript and the incoming NTP. The priming loop remains retracted in both states

To further study conservative transcriptional mechanisms, we imaged DLPs at non-transcribing state with the same methods (Supplementary Figs. 1 and 2 and Supplementary Table 1). In the final sub-particle reconstruction at 3.4 Å resolution, we found no RNA density in the active site; however, two ssRNAs that attach to two separate positions on the surface of RdRp were detected. As detailed below, we interpret that these two ssRNAs are the result of an open genomic duplex. Thus, the RdRps in these DLPs existed mainly in a duplex-open state (DOS) (Fig. 2a) compared to TES (Fig. 2b). With opened duplex and strands outside the active site, this RdRp structure in DLP is different from all previously reported in situ structures of reovirus^18,19,28^. In addition to resolving densities of genomic RNA and mRNA in action, our in situ structures differ from previous rotavirus’s RdRp crystal structures^13^ in the following aspects: we resolved two protein fragments (residues 19–21, 346–358) and identified large conformational changes in three fragments (residues 31–69, 923–996, and 1072–1088) (Supplementary Fig. 4), none of which have been resolved similarly in previous crystallography structures^13,27^. These new structures are essential to understanding the conservative transcriptional mechanism as detailed below.

### RdRp’s N-terminal domain splits the genomic dsRNA

Since only the 3′ end of a single-stranded template can enter the core, the 5′ end of the complementary genomic (+) strand must approach and recognize some region of the RdRp during transcription. In DOS, the cap-binding site of the N-terminal domain (Fig. 2a, c, Supplementary Fig. 5, and Supplementary Movie 4) in RdRp interacts with the conserved terminal m7G(5′)ppp(5′)GGC residues of the genomic (+) strand in all segments of the rotavirus genome (Supplementary Fig. 6). The following bases in all 11 segments of the genome are 6 consecutive bases consisting solely of A and U (Supplementary Fig. 6). In TES, we identified weak densities at the cap-binding site which can only accommodate an NTP molecule (Fig. 2b, d, Supplementary Fig. 5, and Supplementary Movie 4); this cap-binding site has been observed in previous reovirus studies^12,13,18^. Compared with other resolved reovirus RdRp structures^18,28^, the rotavirus RdRp’ N-terminal domain possesses an additional subdomain that has a helix-loop-helix structural feature (residues 31–69, HLH subdomain) near the cap-binding site. This HLH subdomain extends towards the genomic (+) strand in DOS (Fig. 2e) and retracts from RNA in TES (Fig. 2f). The N-terminal domain effectively splits the genome duplex by selectively binding to the 5′-cap-end of the (+) strand RNA, while the HLH subdomain plays a role in further separating the genomic duplex at the downstream AU-box. Later, the (+)RNA bound to the cap-binding site is likely outcompeted by the abundance of NTP in TES.

### RdRp’s core domain polymerizes the complementary RNA

After the dsRNA is split, the unpaired complementary (−)RNA strand traverses the template entrance towards the active site (Fig. 2g–j). In DOS, the (−)RNA weakly interacts with an ssRNA-binding β-sheet subdomain (residues 400–419) in the fingers (residues 333–488, 524–595) of the core, which can bind ssRNA both specifically and nonspecifically^13^. This strand is then guided by this subdomain through a bottleneck towards the palm (residues 489–523, 596–685) in TES (Fig. 2h and Supplementary Fig. 5). A short helix is unwound (residues 398–401) to accommodate the incoming (−)RNA (Fig. 2j), confirming its hypothesized role in mediating template RNA entry^13^. The (−)RNA then immediately pairs with complementary NTP in the active site between the fingers and the palm. The incoming NTPs are in position to form a backbone with the 5′ end of the nascent RNA (Fig. 2j). The priming loop (residues 489–499) is slightly offset between the previously published model^13^ and our atomic models in the two states, but ultimately stays in a retracted position (away from the active site); it is slightly deformed by CSP but remains retracted due to the unexpected refolding of neighboring CSP-B_1_s’ N-terminal arm^27^ (residues 73–92) outside the RdRp (Supplementary Figs. 4 and 7 and Supplementary Movie 5). Thus, the priming loop does not play the suspected stabilizing role^13^ in DOS or TES. Our in situ structure shows that the nascent RNA is first stabilized by two conserved positively-charged residues (K679, R680) in the palm (Supplementary Fig. 7). The RNA then passes by the thumb (residues 686–778), guided by two other conserved residues (R690, R723). No other charge-based interactions are found that influence the nascent RNA. The dsRNA product is then pushed along by the newly-synthesized nascent RNA backbone until it reaches the C-terminal domain.

### RdRp’s C-terminal domain splits the dsRNA product

**Fig. 3:**
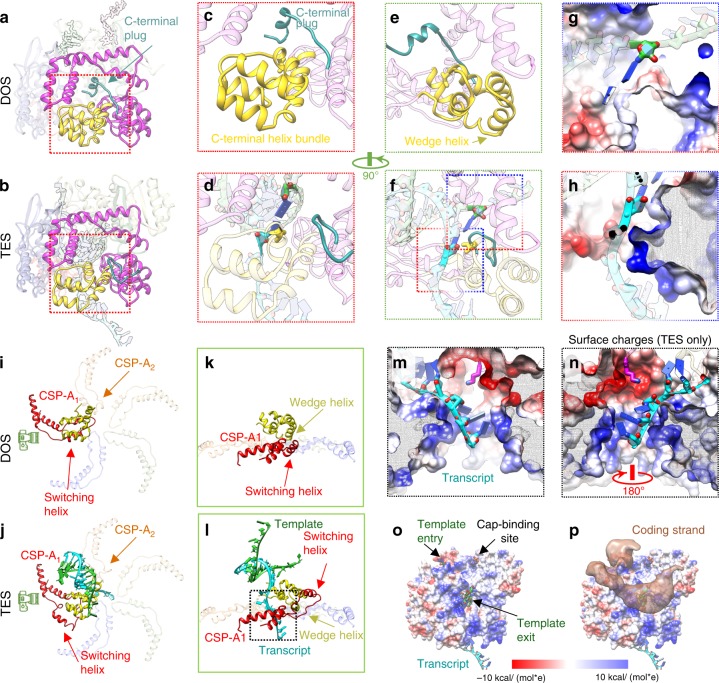
The RdRp C-terminal bracelet domain splits the dsRNA product. **a**, **b** Ribbon models of RdRp’s C-terminal domain (bright) and nearby RNA (silhouetted) in DOS (**a**) and TES (**b**). **c**, **d** Magnified view of the template exit channel in DOS and TES. The C-terminal plug blocks the channel in DOS (**c**). Both the C-terminal plug (dark cyan) and helix-bundle (yellow) undergo dramatic conformational changes in TES (**d**); the helix-bundle is repositioned to aid in duplex base-pair splitting, while the plug is displaced from the exit channel to allow for (−) strand exit. The key residue I944 and the last base pair are highlighted. **e**, **f** 90° turn from the classic front view shows the conformational changes of the helix-bundle and plug in DOS (**e**) and TES (**f**). **g**, **h** Surface charge representations of C-terminal domain regions show that side-chain-induced electric fields guide the RNA backbone and redirect them towards their respective exit channels. Positively-charged surfaces are colored blue and negatively-charged surfaces are colored red. **i**, **j** Ribbon models of RNA and central subdomains within CSP-As in DOS (**i**) and TES (**j**). The transcript can exit a channel through the penton center that is exclusively open in TES. CSP-A_2_’s apical domain remains lifted away from the penton center in both states (orange arrow). **k**, **l** Magnified views from the camera angle in (**i**) and (**j**) show that CSP-A_1_’s apical domain is deformed by the translocated C-HB in TES. **m** Magnified boxed region (penton center) in (**l**) in surface charge representations in TES show that the nascent RNA is flipped by side-chain-induced electric fields at the penton center. **n** A 180° rotation from (**m**) shows the surface charges of the opposite site of the penton center. **o** Surface charge representations of the C-terminal domain show that it forms a highly-positively charged surface region between the template entry channel, cap-binding site, and the template exit channel. **p** Superimposing the coding strand’s density on (**o**) shows that the coding strand density follows the positively-charged RNA track to reanneal with the template. No RNA strand binds to the cap-binding site in TES

For subsequent translation, the RNA transcript must be split from the template prior to its exit through the capsid. Our structure shows key interactions between the C-terminal bracelet domain and the dsRNA product that facilitate this step (Fig. 3a–f). A helix-bundle subdomain (residues 923–996, C-HB) blocks the dsRNA’s trajectory during elongation; specifically, a conserved I944 residue is responsible for disrupting hydrogen bonds, effectively splitting the dsRNA product (Fig. 3d, f). Once separated, bases in both strands are immediately flipped to evade the C-HB, and the negatively-charged backbones are further redirected by side-chain-induced electric fields (SCI-EF) (Fig. 3f–h). As a result, the negatively-charged RNA backbone bends towards the positively-charged surface (blue) and away from the negatively-charged surface (red). The nascent RNA goes towards the capsid through a separate channel between the palm and the bracelet (Supplementary Movie 6). The central subdomain (residues 320–396) of the apical domain (residues 320–596) of five CSP-As is asymmetrically translocated by RdRp (Fig. 3i–l and Supplementary Fig. 8). As a result, a pore is formed through the center of the CSP-A penton (Fig. 3j, l), which processes another SCI-EF to further deflect the nascent RNA (Fig. 3m, n). This nascent RNA eventually exits the capsid shell through this opening in TES. In DOS, however, the C-HB subdomain retracts from CSP-A_1_ and narrows the transcript exit channel (Fig. 3i, k and Supplementary Movie 7), such that CSP-A_1_ returns to a similar conformation as the ones found in CSP-A_3–5_. Two short helixes [residues 349–360 of CSP-A_1_ (switching helix) and residues 968–979 of C-HB (wedge helix)] (Fig. 3k, l) compete for a pocket between CSP-A_1_ and RdRp in these two states. Seeing that no cleaving of peptide chain is involved, this mechanism is likely reversible: the RNA exit channel can be shut after rotavirus’s secondary transcription^29^ and reopened upon entering a new host’s cytoplasm. In contrast, CSP-A_2_’s apical domain remains wedged in both states by the neighboring RdRp (Fig. 3i, j). Simultaneously, the newly isolated (−)RNA exits through the template exit channel located in the center of the C-terminal domain. The C-terminal domain essentially provides a positively-charged ssRNA track on its surface between the template entry and template exit channels; thus, the coding strand can follow this track to reanneal with the template (Fig. 3o, p) and reform the dsRNA genome. The mechanics in the C-terminal domain not only split the dsRNA product (without utilizing additional NTP like other cellular helicases, crucial for conservative transcription), but also redirects the transcript towards the capsid. These movements create sufficient pressure to selectively open a transcript exit channel on demand.

### Two CSP-As’ N-terminal: transcriptional factors

**Fig. 4:**
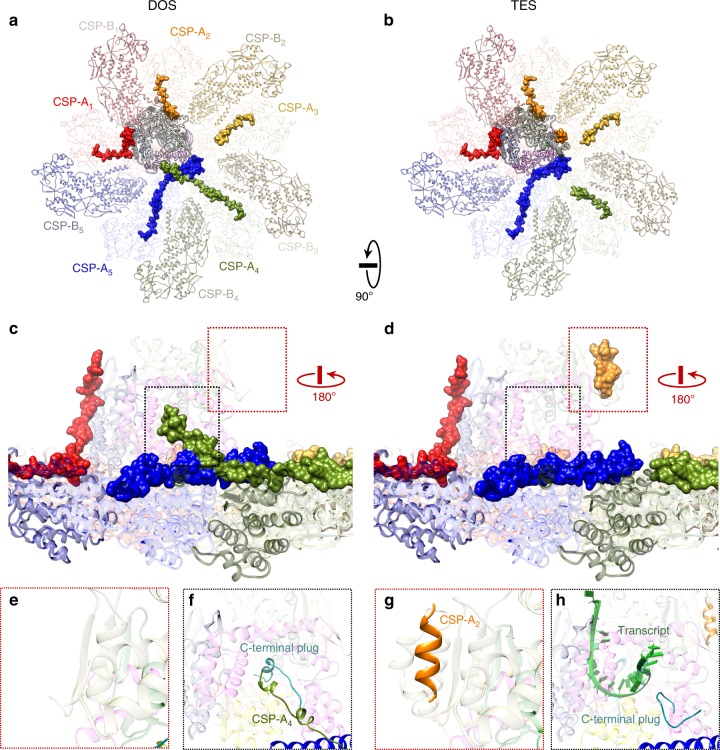
N-terminal of CSP-As form different transcribing complexes with RdRp. **a**, **b** Ribbon diagrams of CSP-As (pale) and RdRp (silhouette). The N-terminals of CSP-As are highlighted with colored surfaces in both DOS (**a**) and TES (**b**). **c**, **d** N-terminal surfaces rotated 90° from the view in (**a**) and (**b**). The binding sites of CSP-A_4_’s (green) and CSP-A_2_’s (orange) N-terminal tethers are boxed in black and red, respectively. **e**–**h** Detailed detachment/attachment of transcriptional factors on RdRp between the two states from the boxed regions in (**c**) and (**d**). The absence of CSP-A_2_’s amphipathic helix in DOS (**e**) and its presence in TES (**g**) suggests that this helix in CSP-A_2_ stabilizes the HLH subdomain in DOS. The presence of CSP-A_4_’s amphipathic helix in DOS (**f**) and its absence in TES (**h**) suggests that this same amphipathic helix in CSP-A_4_ locks C-HB’s conformation in DOS

As a compact nanomachine, rotavirus RdRps also recruit transcriptional factors to regulate their function, similar to other polymerases. CSP-A’s N-terminal regions (residues 62–116) form different transcriptional complexes with RdRp (Fig. 4a–d) through a tethered amphipathic helix (residues 78–84, QLLEVLK, Fig. 4e–h and Supplementary Figs. 9 and 10). This tethered amphipathic helix in CSP-A_2_ attaches to a hydrophobic pocket next to the structured HLH subdomain in TES but detaches from this pocket as the HLH subdomain becomes flexible in DOS (Fig. 4e, g and Supplementary Movie 8). This helix-binding action effectively anchors the HLH subdomain and prevents unfavorable interactions with genomic RNA in TES, thus promoting RdRp activity and RNA release. However, the corresponding amphipathic helix in CSP-A_4_ attaches to the C-HB of RdRp in DOS and detaches from RdRp in TES. The association of this helix closes the template exit channel in DOS and opens it in TES (Fig. 4f, h). In contrast to its counterpart in CSP-A_2_, this helix in CSP-A_4_ actually inhibits RdRp’s activity by locking C-HB’s conformation and blocking the template exit channel. Given these observations, we can conclude that CSP’s N-terminal regions serve as transcriptional regulating factors for RdRp. Similar regulatory mechanisms can also be found in the structure of the rotavirus RdRp itself. A unique C-terminal plug (residues 1072–1088) inserts into the template exit channel in DOS, but moves away in TES to allow (−)RNA to exit. This C-terminal plug is close to the priming loop in DOS and potentially influences the priming loop’s approach to the nascent NTP during initiation (Fig. 2i). Thus, the C-terminal plug is another example of the regulatory factors present in rotavirus transcription/replication. We also find other minority states in our dataset (Supplementary Fig. 11) that potentially reflect the numerous transient states of RdRp.

## Discussion

**Fig. 5:**
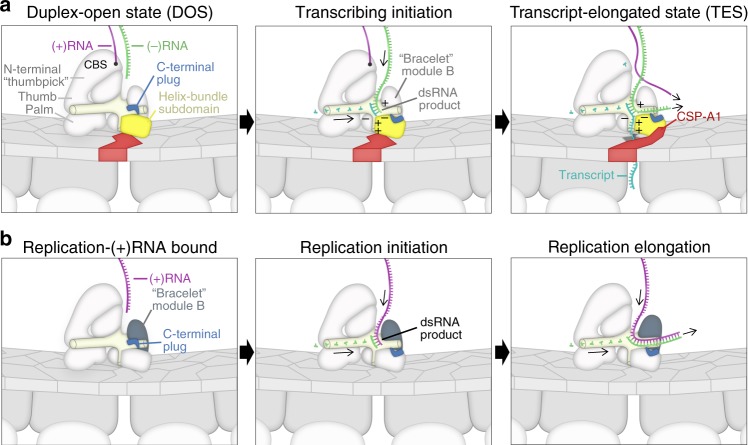
Transcription and replication mechanism. **a** Transcriptional mechanism of rotavirus informed by our in situ RdRp structures in action. **b** Possible mechanism of rotavirus RNA replication, deduced from the observed structures of the transcriptional machinery

Because the N and C terminal domains in rotavirus’ RdRp play such integral roles in its activity, we infer that these may have evolved as critical extensions to the conserved polymerase core (shared by DNA polymerases, telomerases, and RdRp). Both termini effectively function as minimalistic helicases and are essential for conservative transcription. In DOS, the N-terminus is capable of splitting the dsRNA genome with only around 330 residues; this domain recognizes and interacts with 5′ consensus bases (GGC) of (+)RNA at the cap-binding site (CBS), so that the subsequent 6-base-long A/U-only box can be more efficiently split by the neighboring HLH subdomain. As a result, the newly-isolated (−)RNA attaches to the nearby ssRNA recognition site on the fingers (Fig. 2c). This A/U-only region is similar to the A/T-rich TATA box and Pribnow box, which is easily melted and plays a key role in cellular transcription initiation^30^. Because the RdRp’s N-terminal domain interacts with string-like RNA and is close to the thumb, we renamed the N-terminal domain of RdRp the N-terminal “thumbpick” domain. In TES, the C-terminal bracelet not only exhibits functional helicase activity, but also redirects the two RNA strand products to exit through their respective channels. In redirecting RNA strand products, the C-terminal region also helps reorganize the nascent genomes. These peripheral domains allow RdRp to operate in a continuous fashion during transcription (Fig. 5a). In DOS, the 5′ end of genomic (+)RNA binds to CBS, and (−)RNA proceeds to the template entry. The (−)RNA is then transcribed, and the resulting dsRNA product reaches the aforementioned machinery of the C-terminal domain. Specifically, C-HB is needed to split the dsRNA product and isolate the single-stranded transcript. The C-HB subdomain is pushed by the incoming product and realigned to the center of the product’s base pairs in an orientation that allows for effective splitting of the product. As a result, the translocated C-HB subdomain pushes on the CSP-A_1_’s apical domain to selectively open the transcript exit gate on the capsid shell during ongoing transcription. The (−)RNA undergoes a near U-turn (Fig. 4h) in RdRp and returns into the capsid interior near the CBS. Under ideal circumstances [abundance of GTP, accumulation of (+)RNA near CBS], elongation results in the displacement of (+)RNA from CBS by a GTP molecule, allowing (+)RNA to reanneal with the nearby exiting (−)RNA, thus completing the transcription bubble in TES. Intriguingly, we did not find the capping enzyme anchored inside the capsid interior as suspected^25^. Our visualization of the nascent RNA transcript through the CSP shell immediately after exiting from the RdRp exit channel would be consistent with the external location of a capping enzyme lining the 5-fold opening, geometrically similar to its location in turreted reoviruses^31^.

Not only do the N and C terminal domains regulate the genome, but they may also provide interfaces for potential association of transcription factors. This regulation of transcription factors further specializes the protein’s function. In rotavirus, the amphipathic helix in CSP-A_2_ locks the HLH subdomain to prevent further undesirable interactions with the genome during elongation; this same amphipathic helix in CSP-A_4_ locks C-HB and blocks the template exit channel as an inhibiting factor in DOS. This supports previous findings that rotavirus’s RdRp–CSP interactions are crucial for polymerization activity^20,32^. It is also consistent with previous suggestions^28^ that aquareovirus CSP’s N-terminal region can form different transcriptional complexes with the polymerase at different time points.

Understanding the polymorphic nature of the C-terminal domain also yields insights into viral replication (Fig. 5b). Without a complementary strand bound to CBS, the C-terminal domain is less hindered by RNA on its outer surface. When the duplex pushes the C-HB, the upper part of the C-terminal domain (module B) flaps open to let the duplex enter the capsid interior (without the splitting and guiding aspects it displays in transcription), similar to DNA polymerases. This function is recovered in transcription due to both the presence of bound (+)RNA at the beginning of elongation and a relatively crowded capsid interior.

Based on the observation that the capped end of dsRNA leaves RdRp during TES and re-associates with RdRp at cap-binding site in DOS, we propose that the other end of the dsRNA genome (i.e., the tail end) is close to the capped end in DOS. When elongation starts, the entire dsRNA strand is pulled towards the RdRp so that the tail end will leave RdRp, leaving enough space to accommodate the reannealed capped end. At the end of the elongation step, the capped end follows the tail end and circles back to RdRp again. The capped end can then bind to the nearby cap-binding site and start a new transcription cycle, much like an Ouroboros. In this model, the cap is not always bound to the cap-binding site, so there are no undesirable kinks or sharp U-turns on the dsRNA genome during elongation. This model is also more consistent with other RdRps that conduct semi-conservative transcription (e.g., φ6’s RdRp^11^), in which the cap is not bound during transcript elongation. However, φ6 phage’s RdRp differs quite drastically from rotavirus’ in their terminal domains: the RdRp of φ6 has no N-terminal domain, and its C-terminal domain is shorter (65 a.a.) and is suspected to prime polymerization^11^ rather than to split and rearrange RNA products. It is possible that in semi-conservative transcription, the transcript is split from the dsRNA genome by a different mechanism; therefore, in φ6 phage, we do not see N- and C-terminal structures similar to those of rotavirus and other reoviruses that conduct conservative transcription.

In summary, the two in situ structures of rotavirus RNA polymerase in action suggest that the peripheral domains organize RNA for the core, thus acting like up-/down-stream nodes on a specialized production line. Similar to other polymerases, viral RdRps have also evolved their core units to recruit other proteins^18,28^, and we show that the recruited capsid proteins, like cellular transcription factors, form different transcriptional complexes with RdRp. Confined in a crowded viral capsid, the highly specialized rotavirus RdRp has simply co-opted its own N- and C-terminal domains and regions of its capsid protein to regulate transitions between different states. As genome transcription is an essential step in rotavirus infection, the in situ structures presented here, as well as those from others^33^, will also be informative for ongoing drug discovery efforts, in addition to the above-discussed insights about the fundamental biological processes of transcription and replication (Fig. 5).

## Methods

### Double-layered particle purification

Simian rhesus rotavirus (RRV) double-layered particles were purified from rotavirus-infected cells as described elsewhere^34^. Briefly, MA104 cells infected with RRV at a multiplicity of infection (MOI) of 3 were harvested at 100% cytopathic effect. Cell lysate was generated by freezing and thawing twice. The lysate was treated with 50 mM EDTA (pH 8) followed by incubation for 1 h at 37 °C. After centrifugation, the pellet was resuspended in TNC buffer (10 mM Tris–HCl, pH 7.4; 140 mM NaCl; 10 mM CaCl_2_) supplemented with 0.1% Nonidet P-40, and 50 mM EDTA (pH 8) and trichlorotrifluoroethane was added. The aqueous phase was separated by centrifugation, and DLPs were isolated by equilibrium ultracentrifugation at 100,000 × *g* in a CsCl gradient for 18 h. A band containing DLPs was collected, diluted in TNC buffer, and pelleted through a sucrose cushion (15% sucrose prepared in TNC buffer) by ultracentrifugation at 110,000 × *g* for 2 h. Finally, particles were resuspended in 10 mM Tris–HCl, pH 8 prior to either transcription reaction or plunge-freezing.

### Cell-free transcription reaction

For the transcription reaction, purified DLPs were incubated in transcription buffer (10 mM Tris–HCl, pH 8; 4 mM rATP; 2 mM rGTP; 2 mM rCTP and 2 mM rUTP; 0.5 mM S-adenosylmethionine; 6 mM DTT; 9 mM MgCl_2_) for 5 min at 37 °C prior to plunge-freezing for cryoEM.

### CryoEM and 3D asymmetric reconstruction by symmetric relaxation

An aliquot of 2.5 μl of each sample was applied to plasma-cleaned Quantifoil 1.2/1.3 holey cryoEM grids, which were blotted and plunge-frozen with an FEI Vitrobot Mark IV.

High quality cryoEM images were then collected in an FEI Titan Krios 300 kV electron microscope, equipped with a Gatan K2 direct electron detector and a Gatan Quantum energy filter. The microscope was carefully aligned and the coma-free alignment was performed to align the beam tilt immediately before the data collection. As detailed in Supplementary Fig. 2, we collected both data sets using the counting mode at a frame rate of 8 frames per second without putting in the slit of the energy filter with LEGINON^35^ automation. DOS data was collected for 8 s with a calibrated pixel size of 1.07 Å, while TES was collected for 10 s with a calibrated pixel size of 1.33 Å. The first 25 frames in DOS and first 32 frames in TES were aligned with UCSF MotionCorr software^36^ to make micrographs with 22e per Å^2^ and 18e per Å^2^ dosages, respectively. Contrast transfer function (CTF) parameters were determined with CTFFIND4^37^ for both datasets.

For DOS, particles were automatically boxed with ETHAN^38^. Virus particles’ center and orientations were refined with Relion^39^ with icosahedral symmetry i2 (i.e., the convention with *x*, *y*, and *z* axes along the icosahedral 2-fold axes) applied. The final resolution of the resulting icosahedral reconstruction at FSC > 0.143 is 3.6 Å.

To obtain the asymmetric structure of the polymerase, we conducted localized reconstruction^25^ to focus on particle vertices (i.e., each vertex treated as a sub-particle). First, the icosahedral reconstruction is rotated to follow an icosahedral symmetry i3 (5-fold axis aligned with *z* axis) and particle orientations were adjusted accordingly^39^ (Supplementary Fig. 2I). For each particle in the dataset, we calculated the coordinates (rlnOriginX and rlnOriginY) and orientation parameters for the 12 sub-particles (vertices) using a Python script^40^. These coordinates were then used to box out sub-particles by the relion_preprocess command from the RELION package^39^. Second, each sub-particle was then expanded with the “relion_particle_symmetry_expand” command as 5 entries (Supplementary Fig. 2II) in the RELION star file, each having a 5-fold-related orientation around the *z* axis (i.e., only rotational Euler angle (_rlnAngleRot) differs from each other by an increment of 72°). Third, all sub-particles were then subjected to RELION 3D classification by asking for 16 classes with the “skip_align” option (III in the left panel of Supplementary Fig. 2) resulting in 9 “good” classes (i.e., those with densities that can be interpreted as one single RdRp at certain density threshold and are demarcated with color arrows in III of Supplementary Fig. 2) and 7 bad classes (colored in cyan in Supplementary Fig. 2). These 9 good classes can be further grouped into 5 groups (colored red, orange, green, blue, and purple for group A, B, C, D, and E, respectively in Supplementary Fig. 2) based on the orientation of the RdRp in the reconstruction of each good class, while the 7 bad classes were grouped into group X. In the ideal situation, the five consecutive entries of every sub-particle should be sequentially placed into one of the five circular permutations of group list A, B, C, D, and E. However, our observed results (Supplementary Fig. 2, step III) deviated from such ideal situation for two possible reasons: First, the 5-fold-related capsid proteins could have obscured the alignment signals during classification; Second, there might be multiple conformations of RdRp.

To make the optimal group placement choice for the sub-particles based on our observed results, we developed a Python script program (Orientation_Selection.py) that processes the RELION star file. By taking the star file from 3D classification as input, this script analyzed order of group (A, B, C, D, E, or X) placements of the five entries of each sub-particle and find its best match to the 5 possible circular permutations of the ideal group list. If the best match has less than two outliers out of the five groups, this sub-particle will be retained with permuted orientation; otherwise, this sub-particle will be discarded. For example, the result group list “B,C,D,X,A” best matches one-time permuted ideal list “B,C,D,E,A” with one outlier so this sub-particle would be retained with one rotation of 72°, but result group list “B,C,D,X,E” matches permuted ideal list “B,C,D,E,A” with two outliers so this sub-particle would be discarded. A new star file was created with the retained sub-particle and their orientation assignments. A RELION local classification with limited range of angle search (relion parameter--sigma_ang 3) was then performed to select the major conformation (Supplementary Fig. 11). A RELION gold-standard local refinement was finally conducted and the final sub-particle reconstruction reached 3.4 Å resolution (step IV in Supplementary Fig. 2).

For TES, we used a similar method as stated above. The resolutions for the icosahedral reconstruction is 3.4 Å and that for the vertex sub-particle reconstruction is 3.6 Å (Supplementary Fig. 2V–VIII).

### Atomic model building and model refinement

The atomic models of RRV’s RdRp and CSP were built with Coot^41^ and refined with Phenix^42^. We first used the “fit in map” function of UCSF Chimera^43^ to dock PDB 4F5X, a previously published montage model, into the sub-particle reconstructions of the two states. There are six kinds of major discrepancies: previously flexible regions in crystallography (residues 19–21, 346–358 in RdRp); backbone tracing error (residues 804–821 in CSP); newly-resolved asymmetric features (residues 62–117, 336–373 in CSP-A); conformational changes introduced by RdRp’s docking on CSP (residues 487–510 in RdRp, 73–93 in CSP-B_1_); large conformational changes between different states (residues 31–69, 923–996, 1072–1088 in RdRp); and in situ RNA features (the template, transcript, coding strand, and NTP). For those discrepancies, we manually traced the backbone in all-alanine mode in Coot and then mutated them into the correct sequence. RNA in DOS was built with conserved sequences m7GpppGGC at the 5′ end of the coding strand and its complementary strand, while RNA in TES was built with repetitive AU polynucleotides. The models in both states were then refined by the PHENIX real-space refine function and validated by the wwPDB validation server^44^.

Visualization of the atomic model, including figures and movies, is made with UCSF Chimera^43^. The sequence is visualized by ESPRIPT^45^.

### Reporting summary

Further information on research design is available in the Nature Research Reporting Summary linked to this article.

## Supplementary information

Supplementary Information

Description of Additional Supplementary Files

Supplementary Movie 1

Supplementary Movie 2

Supplementary Movie 3

Supplementary Movie 4

Supplementary Movie 5

Supplementary Movie 6

Supplementary Movie 7

Supplementary Movie 8

Reporting Summary

## Data Availability

The cryoEM density maps have been deposited in the Electron Microscopy Data Bank under accession codes EMD-20059 (DOS) and EMD-20060 (TES). The atomic coordinates have been deposited in the Protein Data Bank under accession codes 6OGY (DOS) and 6OGZ (TES). Other data are available from the corresponding authors upon reasonable request.

## Code availability

Custom-designed programs for particle extraction and orientation selection are deposited in https://github.com/kerichardding/Rotavirus_scripts.
