# Supplementary material for: In situ structures of rotavirus polymerase in action and mechanism of mRNA transcription and release
Source: Nat Commun. 2019 May 17;10:2216. doi: 10.1038/s41467-019-10236-7 (PMC6525196; doi:10.1038/s41467-019-10236-7)
Supplement: Supplementary file 1 — Supplementary Information [file 41467_2019_10236_MOESM1_ESM.pdf]

In situ structures of rotavirus polymerase in action and mechanism of mRNA transcription and release

Ding et al.

Supplementary Information

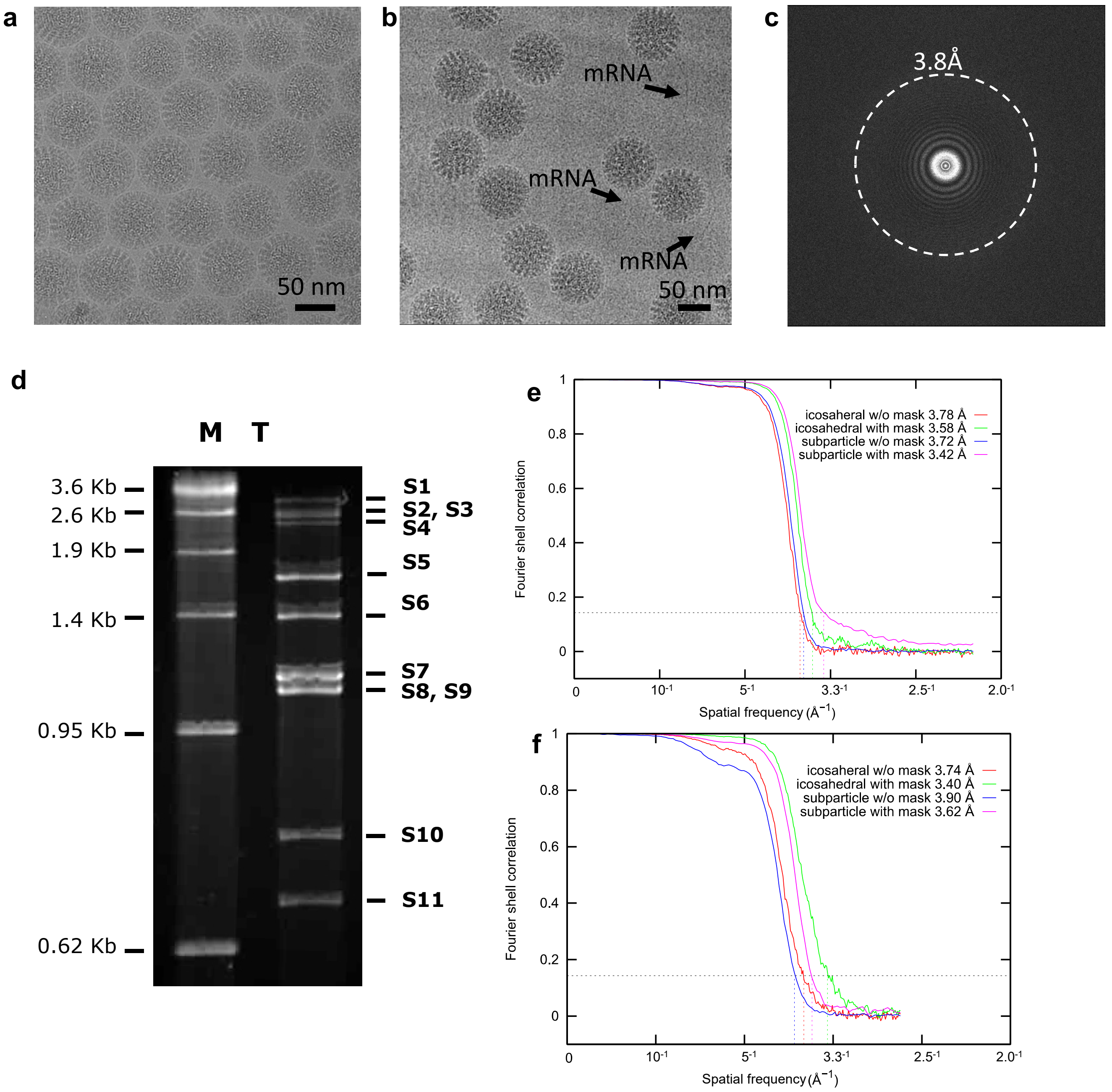

Supplementary Figure 1 CryoEM and structure determination statistics.
a, b, CryoEM images of rotavirus DLP (**a**) and transcribing DLP (**b**). **c,** Fourier transform of a micrograph of rotavirus DLP, to show the signal limit. **d,** 4% acrylamide- 8M Ura gel to show the transcription product of DLP's *in vitro* transcription. Lane M: single-stranded RNA marker. Lane T: in vitro transcripts obtained from DLPs. **e, f,** Fourier shell correlation (FSC) coefficient as a function of spatial frequency of DLP (**e**) and transcribing DLP (**f**) reconstructions.

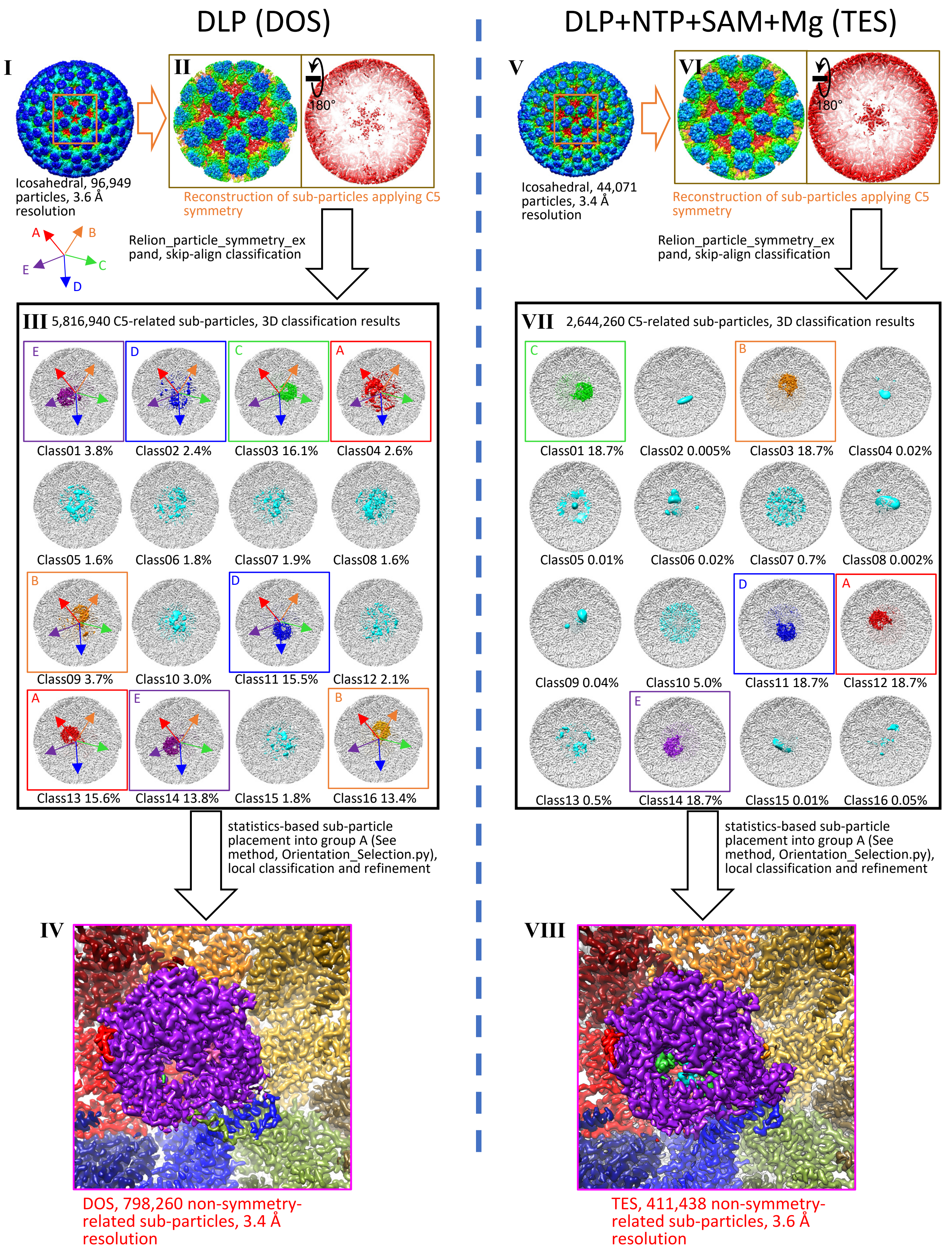

Supplementary Figure 2 Data processing workflow for sub-particle reconstructions of DLP (left) and transcribing DLP (right).

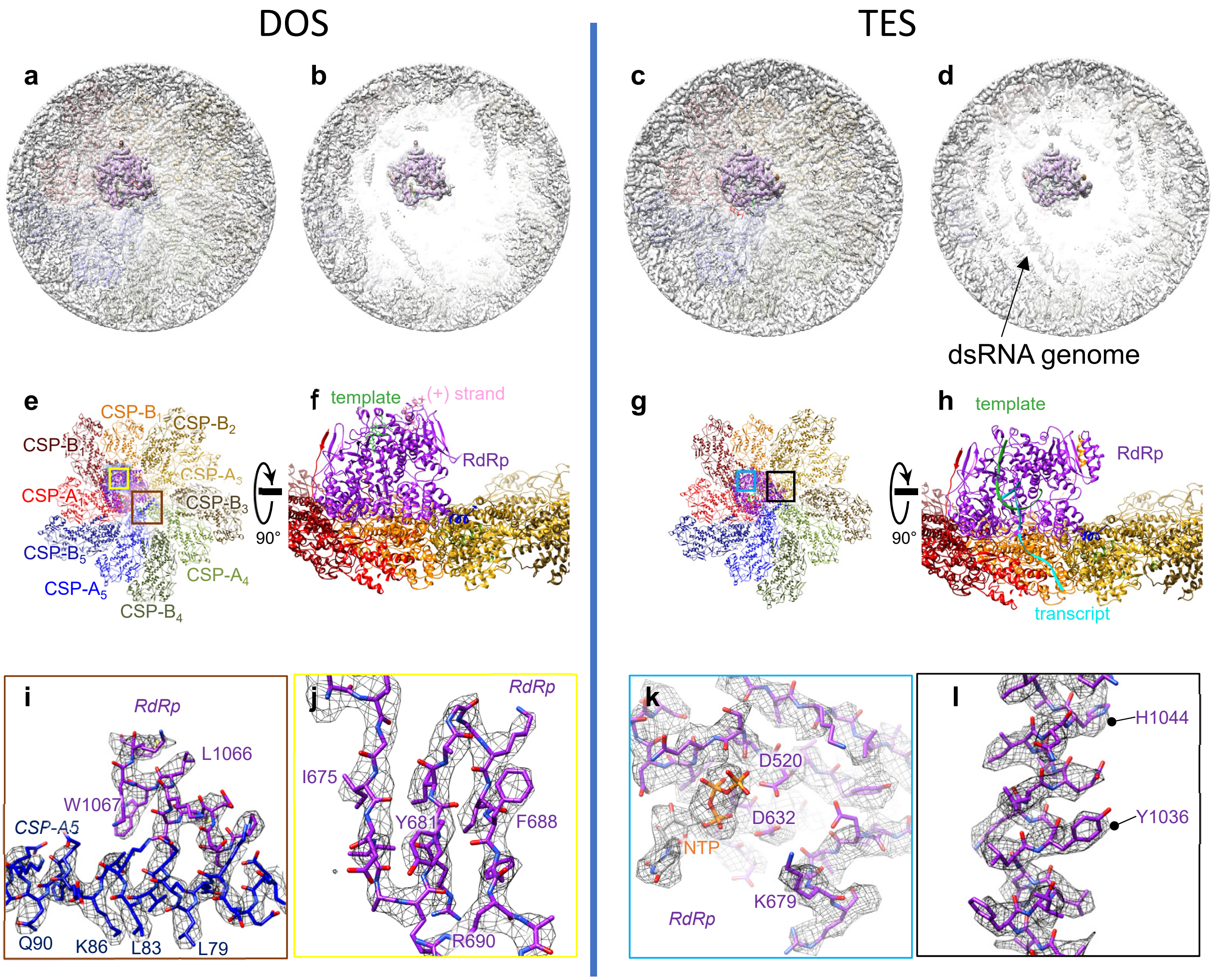

Supplementary Figure 3 Sub-particle reconstructions and atomic models of RdRp with associated CSP decamer in two states.

a, b, Internal surface view of the DOS sub-particle reconstruction shown fully (**a**) and partially without the rear portion (**b**). **c, d,** Same as (**a, b**) but in TES. Note the surrounding RNA features are better resolved in TES than in DOS. **e-h,** Ribbon diagram of the atomic models of RdRp (purple) and CSP (rainbow) in DOS (**e, f**) and in TES (**g, h**) in two orthogonal views. **i-l** Densities (wires) in the boxed regions of (**e, g**), superposed with atomic models, highlighting some high-resolution features in our structures, including an interaction between RdRp and CSP-A_5 in DOS (**i**), a β-sheet in RdRp in DOS (**j**), the active site with an NTP in TES (**k**), and a helix bundle in RdRp in TES (**l**). See also Supplementary Movie 1.

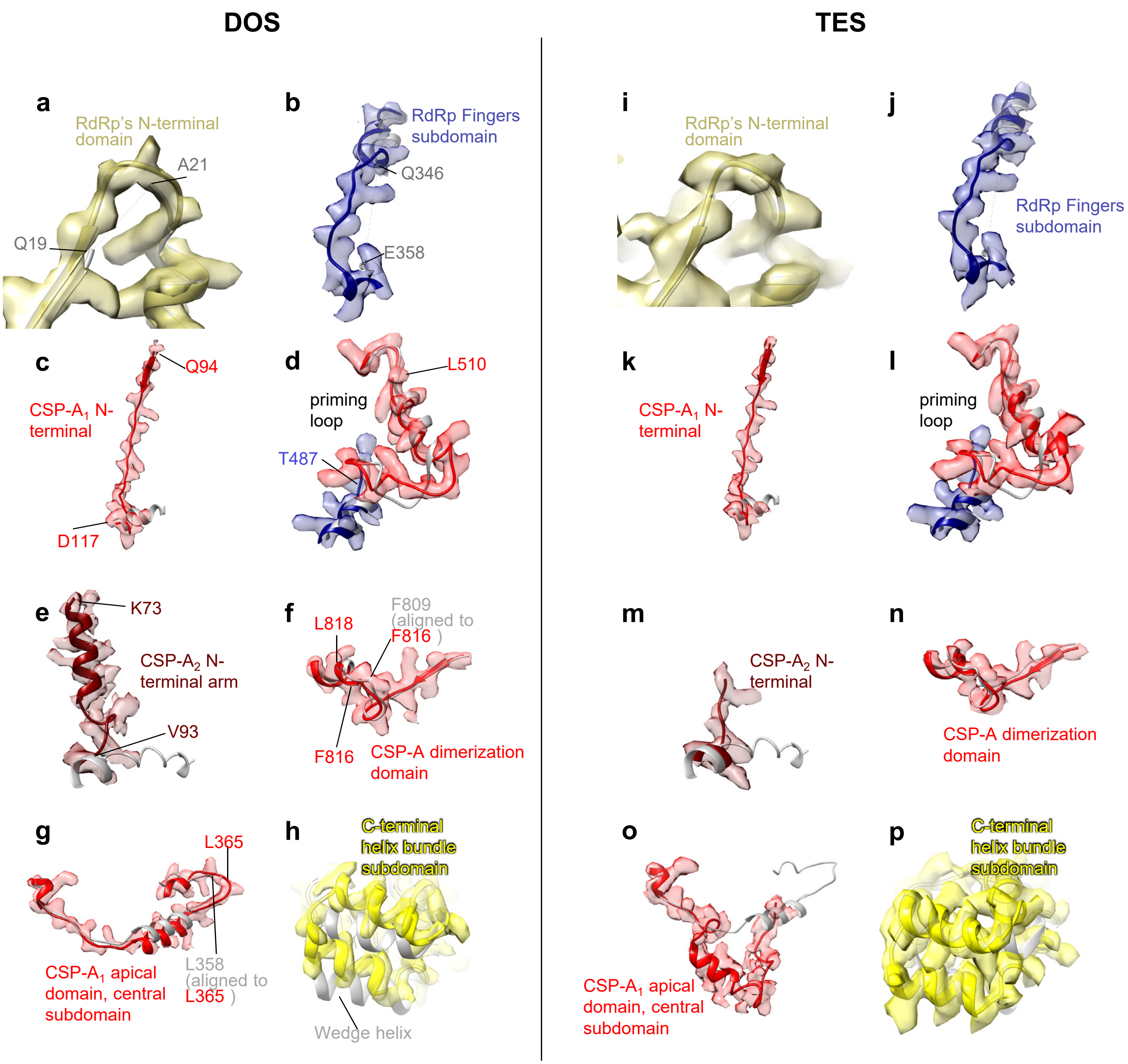

Supplementary Figure 4 Key differences between our models and the previous model.

Our atomic models (color ribbons) are superposed on the cryoEM density map (semi-transparent) for various fragments (residues indicated) of RdRp and various CSP-A conformers in both DOS (**a-h**) and TES (**i-p**). For comparison, the old model (PDB 2R7Q for RdRp and PDB 4F5X for the capsid) are all shown in grey ribbons, though no previous model existed for the most of the panels shown here.

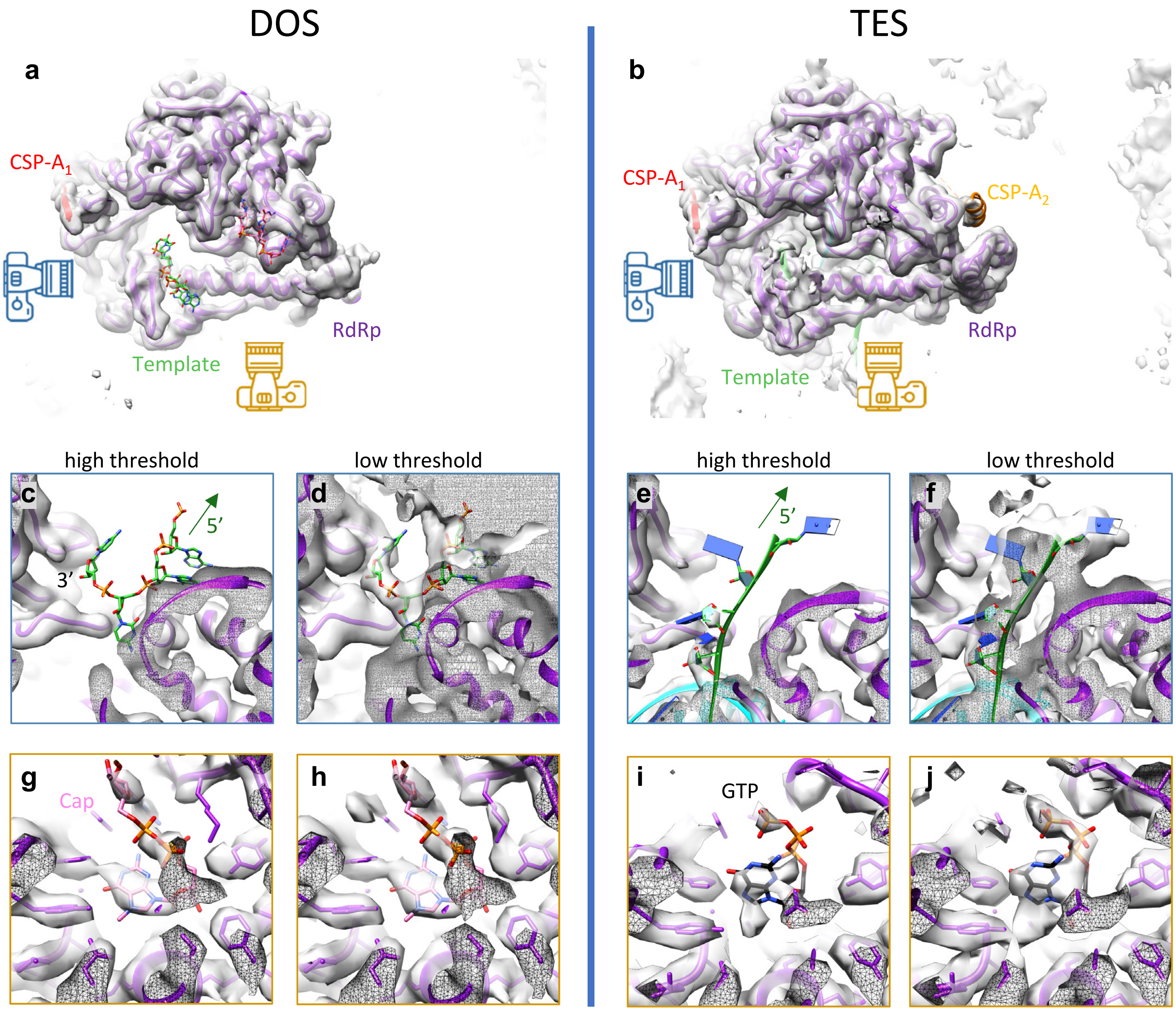

Supplementary Figure 5 Template entry and cap-binding site.
a, b, RdRp in DOS (**a**) and TES (**b**), shown in the classic top view with models of RdRp (purple) and RNA (ball-and-stick) fit in the Gaussian-filtered map with a 2 Å edge. The camera symbols depict the viewing directions for panels shown in **c-j**. **c-f,** Magnified views (blue camera in **a** and **b**) of the template entrance in DOS (**c**, **d**) and TES (**e**, **f**) at different density thresholds. The template RNA has more flexibility towards 5’ end and some features can only be seen at a low density thresholds in the filtered density map. **g-j,** Magnified views (orange camera in **a** and **b**) of the cap-binding site in DOS (**g**, **h**) and TES (**i**, **j**) at two different density thresholds. In DOS, the cap-binding site binds the m7GpppG cap of the (+)RNA strand with high affinity. In TES, the cap-binding site binds a GTP and the feature can be better seen at a lower display threshold. See also Supplementary Movies 1 and 4.

```
             1.......... .......... ..........        3271.......... .......... .......... ..
EU636924.1    ggctattaaa gctgtacaat ggggaagtat            ttctttcaag attagaacgc ttagatgtga cc
            31.......... .......... ..........
              aatctaatct tgtcagaata tttatcattc
```

```
VP2          1.......... .......... ..........        2671.......... .......... .......... ........
EU636925.1    ggctattaaa ggctcaatgg cgtacagaaa            cgaactgtaa acgccaaccc cattgtggag atatgacc
            31.......... .......... ..........
              gcgtggagcg cgtcgtgaga cgaatttaaa
```

```
VP3          1.......... .......... ..........        2551.......... .......... .......... .......... .
EU636926.1    ggcttttaaa gcagtaccag tagtgtgttt            tgagtgagct agaaacttaa cacactagtc atgatgtggc c
            31.......... .......... ..........
              tacctctaat ggtgtaaaca tgaaagtact
```

```
VP4          1.......... .......... ..........        2331.......... .......... .......... ..
EU636927.1    ggctataaaa tggcttcgct catttataga            tagactgtaa gcaatttcca gaggatgtga cc
            31.......... .......... ..........
              caattgctta caaattcata taccgttgac
```

```
NSP1         1.......... .......... ..........        1511.......... .......... .......... .......... ..........
EU636928.1    ggcttttttt atgaaaagtc ttgtgttagc            gactaatgat tgaattaact atcaccacag tttttgccat cacaagacct
            31.......... .......... ..........        1561.......... .......... .......... .......... .
              catggcaacc tttaaggatg cttgctttca            tctggactag agtagcgcct agctggcaaa aaatgtgaac c
```

```
VP6          1.......... .......... ..........        1211.......... .......... .......... .......... ..........
EU636929.1    ggcttttaaa cgaagtcttc aacatggatg            caaatgagga ccaagctaac cacttggtat ccgactttga tgagtatgta
            31.......... .......... ..........        1261.......... .......... .......... .......... ..........
              tcctgtactc cttgtcaaaa actcttaaag            gcttcgtcaa gctgtttgaa ctctgtaagt aaggatgcgt ccacgtattc
                                                      1311.......... .......... .......... .......... ......
                                                          gctacacaga gtaatcactc agatggtata gtgagaggat gtgacc
```

```
NSP3         1.......... .......... ..........         961.......... .......... .......... .......... ..........
EU636930.1    ggcatttaat gcttttcagt ggttgatgct            tgagtaattg aatgaacaat tcaatactat taccatctac acgtaaccct
            31.......... .......... ..........        1011.......... .......... .......... .......... ..........
              caagatggag tctactcagc agatggcttc            ctatgagcac aatagttaaa agctaacact gtcaaaaacc taaatggcta
                                                      1061.......... ........
                                                          taggggcgtt atgtgacc
```

```
NSP2         1.......... .......... ..........         991.......... .......... .......... .......... ..........
EU636931.1    ggcttttaaa gcgtctcagt cgccgtttga            aggaatttaa ttcgttatca atttgagagt gggtatgaca aagtaagaat
            31.......... .......... ..........        1041.......... .........
              gccttgcggt gtagccatgg ctgagctagc            agaaagcgct tatgtgacc
```

```
VP7          1.......... .......... ..........        1021.......... .......... .......... .......... ..
EU636932.1    ggctttaaaa gcgagaattt ccgtttggct            agaatatagg tatagctttg gttagaattg tatgatgtga cc
            31.......... .......... ..........
              agcggttagc tccttttaat gtatggtatt
```

```
NSP4         1.......... .......... ..........         561.......... .......... .......... .......... ..........
EU636933.1    ggcttttaaa agttctgttc cgagagagcg            tcattgtgag aggttgagct gccgtcgtct gtctgcggaa gcggcggagt
            31.......... .......... ..........         611.......... .......... .......... .......... ..........
              cgtgcggaaa gatggaaaag cttaccgacc            tcttaacagt aagccccatc ggacctgatg actggttgag aagccacaac
                                                       661.......... .......... .......... .......... ..........
                                                          cagtcatatc gcgtgtgact cagtcttaat cccgtttaac caatccagcc
                                                       711.......... .......... .......... ..........
                                                          agcgctggac gttaatggaa ggaacggtct taatgtgacc
```

```
NSP5         1.......... .......... ..........         611.......... .......... .......... .......... ..........
EU636934.1    ggcttttaaa gcgctacagt gatgtctctc            atttgtaagt ctaacctgag gactcactag gaagctcccc actccagtta
            31.......... .......... ..........         661.......
              agtattgacg tgacaagtct tccatctatt            tgtgacc
```

Supplementary Figure 6 The 5' and 3' fragments of the 11 genome segments of RRV strain A. The 5' consensus sequence is coloured blue. A/U box is underlined. Start and stop codons are coloured red. The 3' consensus sequence is coloured green.

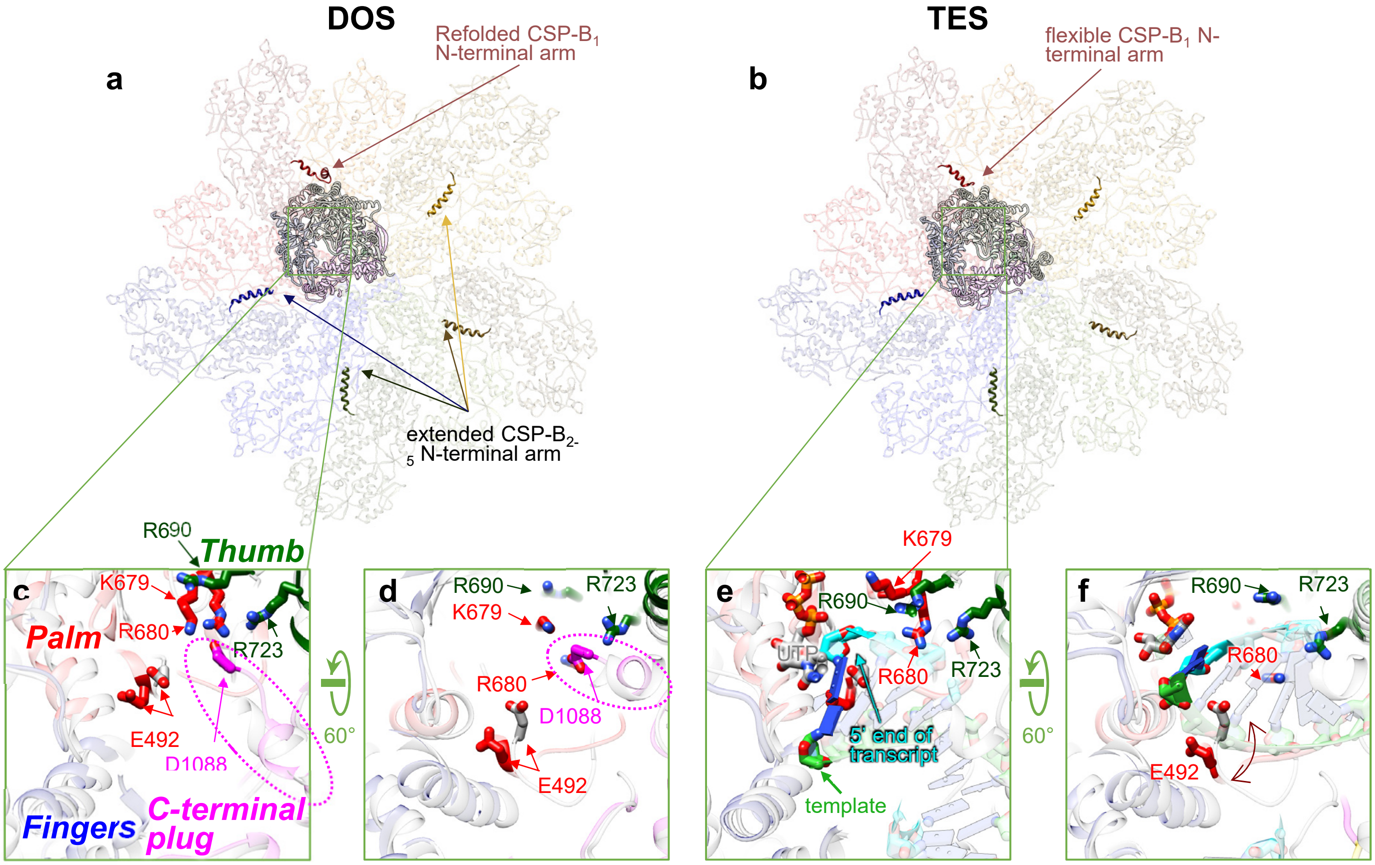

Supplementary Figure 7 RdRp's active site and the "priming loop".

a, Classical top view of RdRp and CSP decamer at DOS. The 5 N-terminal "tethered arms" (one each from CSP-$B_{1\text{-}5}$) are highlighted. **b,** Classical top view of RdRp and CSP decamer at TES. **c, d,** Magnified active site in (**a**)**.** The view in (**d**) is rotated 60-degrees from the one in (**c**)**.** A model from crystallography structure (PDB 2R7R, coloured grey) is superimposed. The key residue (E492) on the "priming loop" is labeled to show that the "priming loop" is not extended towards the active site when the RdRp is docked on the capsid. Also, the N-terminal plug (circled, colored in magenta) is inserted nearby. **e, f,** The same views that (**c**) and (**d**) depict, for TES. The C-terminal plug is retracted from the active site while the "priming loop" remains folded at TES. See also Supplementary Movie 5.

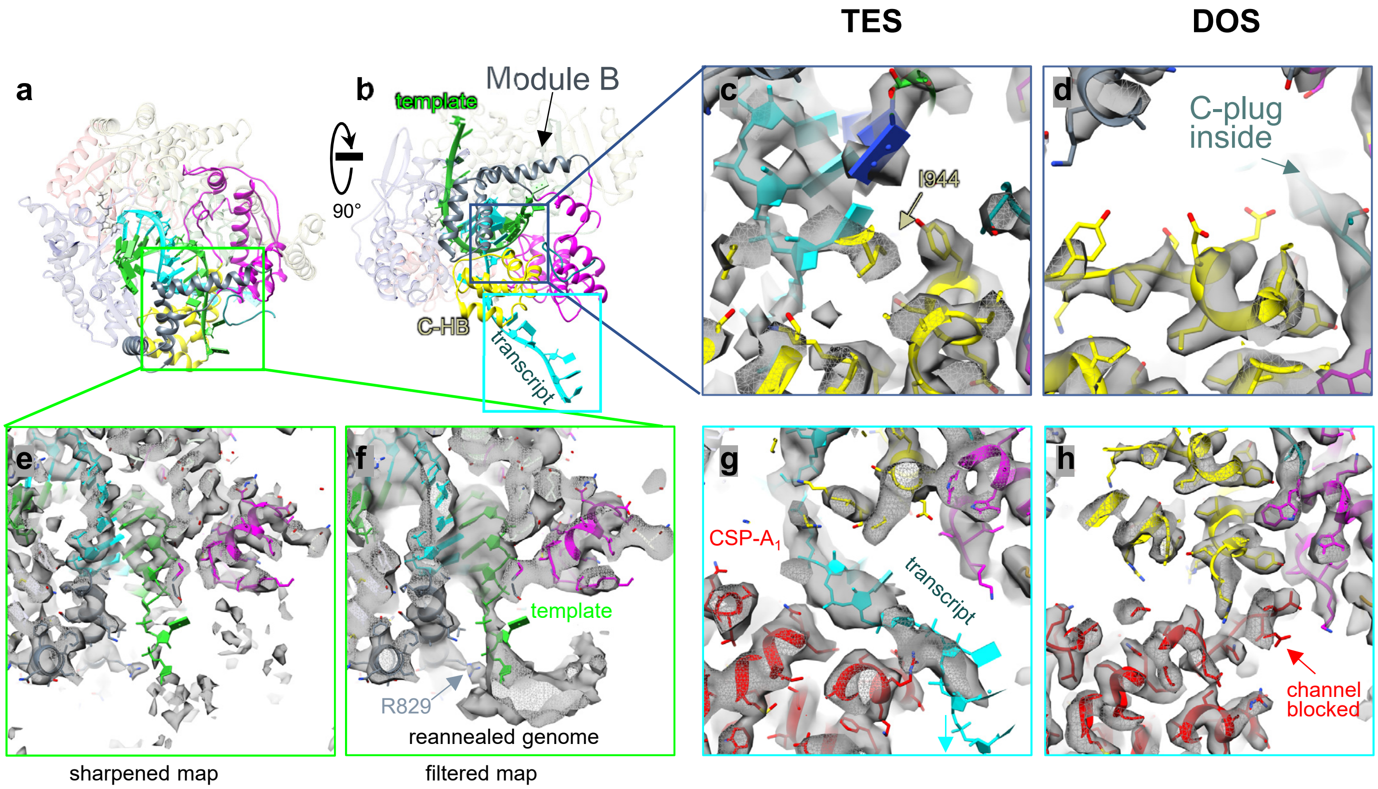

Supplementary Figure 8 Quality of the cryoEM densities of RdRp C-terminal domain and RNA in the template and transcript exit channels.

a, b, Two orthogonal views of the atomic model of RdRp together with transcript and template RNA strands at TES. **c,** Magnified from the boxed region in (**b**). Helix bundle subdomain in the C-terminal domain (C-HB) splits the dsRNA product at TES with I944. **d,** C-HB is retracted, and the C-terminal plug is found inside the template exit channel at DOS. **e, f,** Details around the template exit in a sharpened map (**e**) and in a low-pass-filtered map (**f**) showing the template strand's reannealing with the coding strand. **g, h,** Details around the transcript exit in TES (**g**) and DOS (**h**), with the transcript leaving the capsid unobstructed in TES and a CSP-A_1 loop blocking the channel in DOS. See also Supplementary Movie 6.

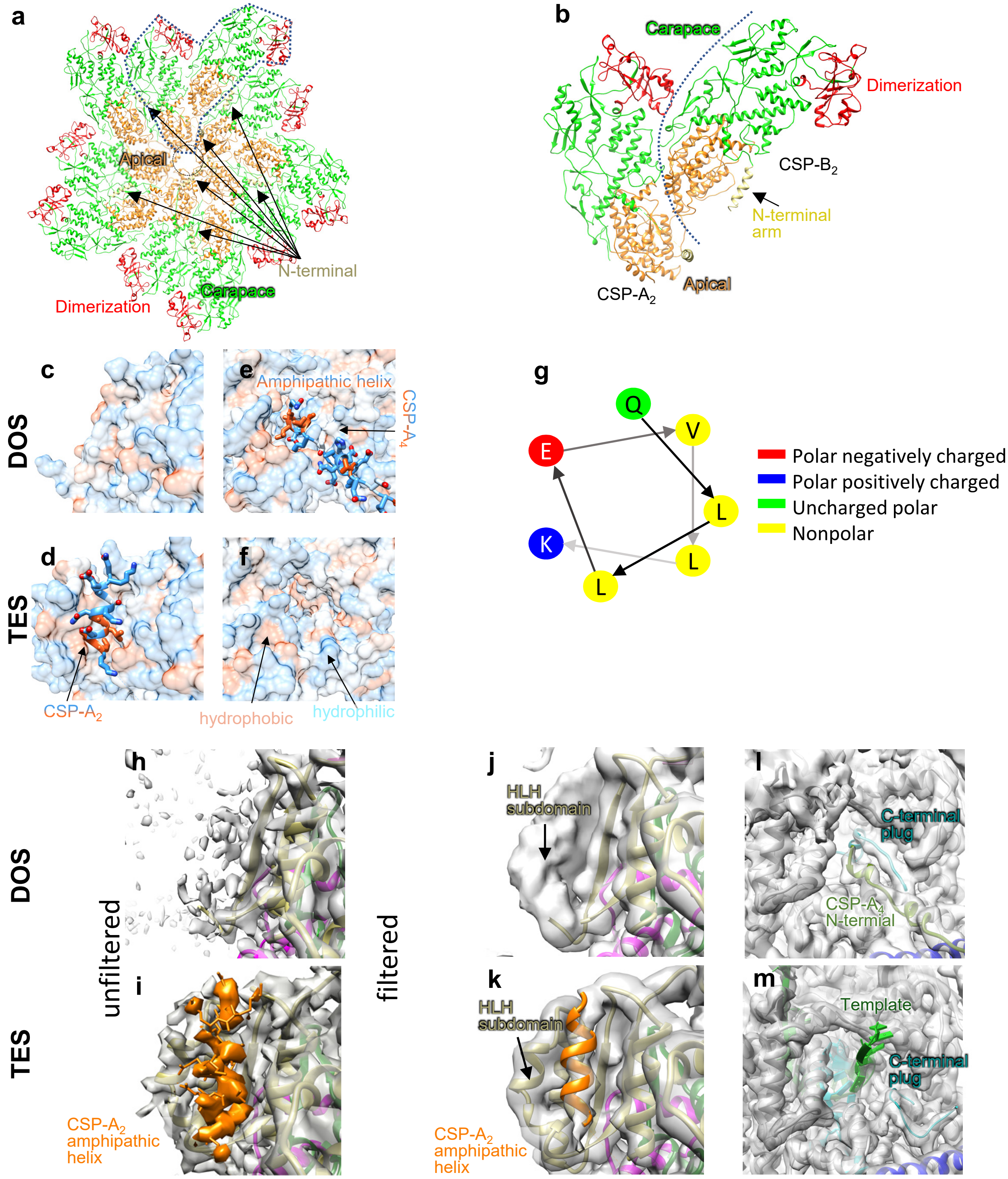

Supplementary Figure 9 CSPs and their N-terminal amphipathic helixes acting as transcriptional factors.
a, An overview of CSP decamer at TES. Unlike the decamer colour-coded by subunits shown in Fig. 4**a**, the current display is colour-coded by domains such that the N-terminal domain of CSP and the open hole (circled) at TES can be easily seen. **b,** Magnified CSP-A_2 and CSP-B_2 from (**a**). **c, d,** Comparison of the N-terminal domain of RdRp between DOS (**c**) and TES (**d**), showing that CSP-A_2's amphipathic helix binds to a hydrophobic pocket that exists only in TES. This pocket is formed by the helix-loop-helix subdomain at TES. In DOS, this subdomain is flexible and no such hydrophobic pocket exists. **e, f,** Comparison of the C-HB domain of RdRp between DOS (**e**) and TES (**f**), showing that CSP-A_4's amphipathic helix only binds to C-HB and blocks the template exit channel at DOS. This part of the structure no longer interacts with C-HB at TES and C-HB is translocated. **g**, Wheel displaying the amphipathic nature of the CSP N-terminal helix. **h-m,** Detailed detachment/attachment of transcriptional factors on RdRp between the two states, shown with corresponding densities, unfiltered (**h,i**) and filtered (**j-m**). The absence of CSP-A_2's amphipathic helix in DOS (**h,j**) and its presence in TES (**i,k**) suggests that this helix in CSP-A_2 stabilises the HLH subdomain in DOS. The presence of CSP-A_4's amphipathic helix in DOS (**l**) and its absence in TES (**m**) suggests that this same amphipathic helix in CSP-A_4 locks C-HB's conformation in DOS. See also Supplementary Movie 8.

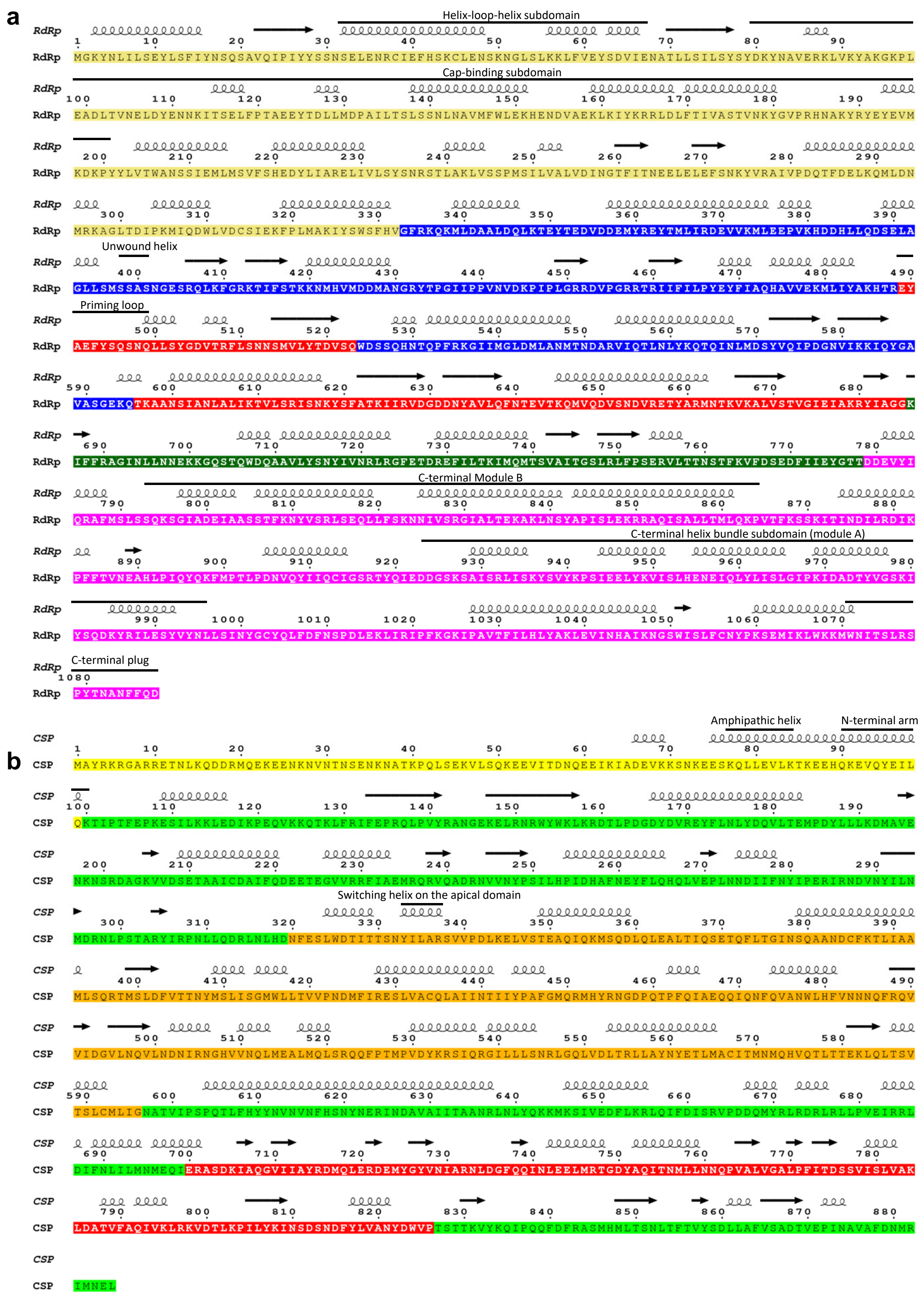

Supplementary Figure 10 Secondary structures and domain assignments of RdRp and CSP.

a, Sequences of RdRp (**a**) and CSP (**b**) along with their secondary structures determined from our cryoEM structures. The sequence is colour-shaped according to domain colours as indicated. See also Supplementary Movie 3 for coloured domains of RdRp.

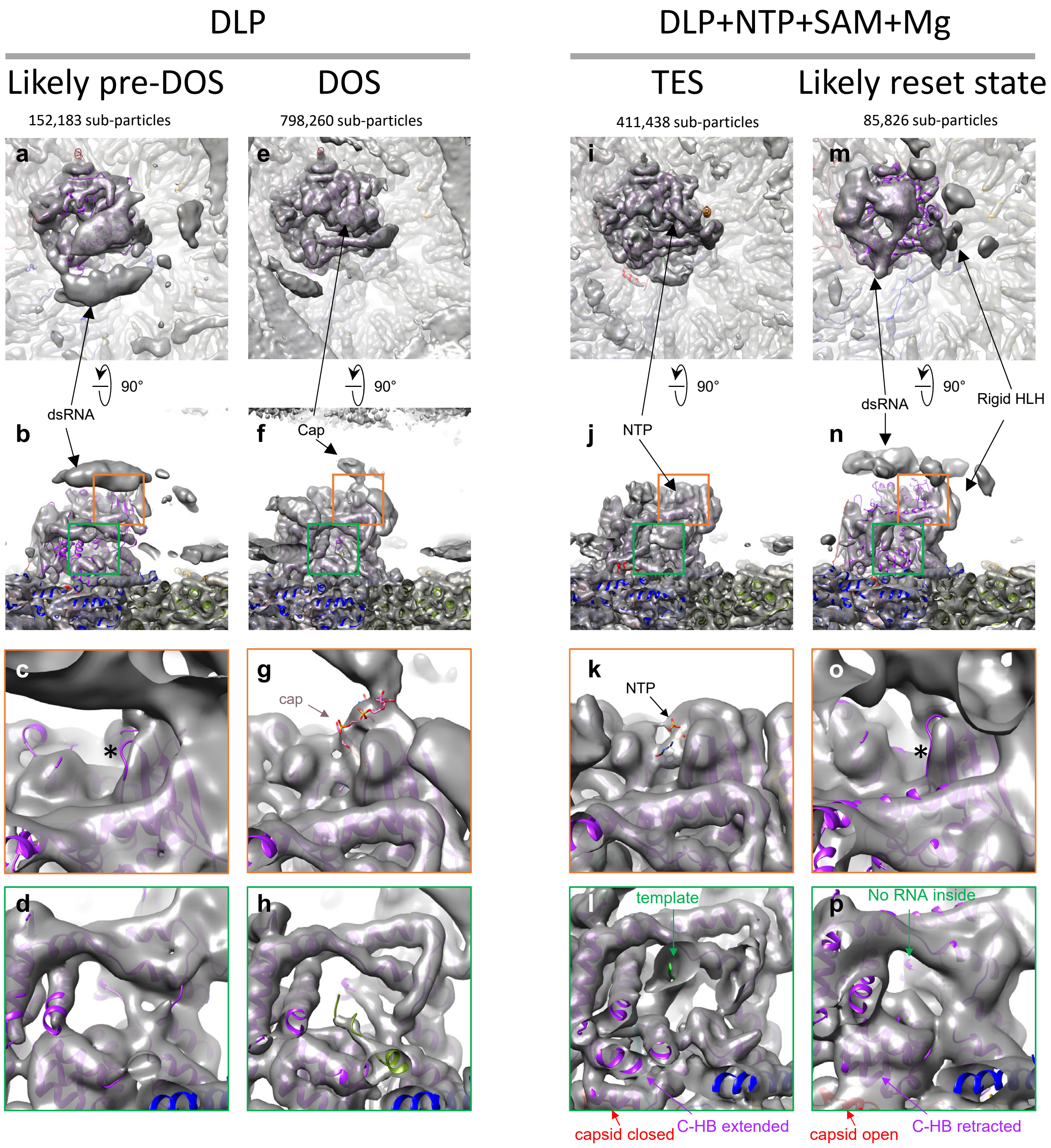

Supplementary Figure 11 Identification of other possible states.

a, b, Two orthogonal views of a reconstruction (with atomic model superimposed) from classified particle in DLP, likely a pre-DOS. **c, d,** Magnified cap-binding site (**c**) and template exit channel (**d**) in (**b**). In this state, the RNA remains in the duplex form and there is no NTP/CAP in the cap-binding site [* in (c)]. **e-h,** The same views as (**a-d**), showing corresponding views at DOS. **i-l,** The same views as (**a-d**), showing corresponding views at TES. **m-p,** The same views as (**a-d**), showing corresponding views at a state different from TES. This state is likely a reset state. In the putative reset state, there is no RNA density inside RdRp (**p**); the C-terminal helix bundle subdomain of RdRp is retracted from the capsid; the C-terminal plug of RdRp is inserted into the active site; and no NTP/RNA bound to the cap-binding site [* in (**o**)]. Notably, though no NTP/RNA are bound to the cap-binding site, a bulky dsRNA genome density [indicated in (**m**) and (**n**)] similar to that [indicated in (**a**) and (**b**)] also seen in pre-DOS, suggests that this complex is *reset* to start the transcription again.

Cryo-EM data collection, refinement and validation statistics

	RdRp in DOS (EMDB-20059) (PDB 6OGY)	RdRp in TES (EMDB-20060) (PDB 6OGZ)
**Data collection and processing**		
Magnification	130K	105K
Voltage (kV)	300	300
Electron exposure (e–/Å^2)	22	18
Defocus range (μm)	1.4-5.4	1.2-5.4
Pixel size (Å)	1.07	1.33
Symmetry imposed	C1	C1
Initial particle images (no.)	1163388	528852
Final particle images (no.)	798260	411438
Map resolution (Å)	3.4	3.6
FSC threshold 0.143		
Map resolution range (Å)	∞-3.4	∞-3.6
**Refinement**		
Initial model used (PDB code)	2R7R	2R7R
Model resolution (Å)	3.45	3.63
FSC threshold 0.143		
Map CC	0.8558	0.8616
Model resolution range (Å)	--	--
Map sharpening *B* factor (Å^2)	-160	-160
Model composition		
Non-hydrogen atoms	74364	74962
Protein residues	9081	9081
Nucleotide residues	6	35
Ligands	1	2
B factors (Å^2)		
Protein	56.83	26.96
Nucleotide	131.39	85.24
Ligand	99.28	60.59
R.m.s. deviations		
Bond lengths (Å)	0.006	0.008
Bond angles (°)	0.767	0.882
Validation		
MolProbity score	1.41	1.59
Clashscore	3.56	5.44
Poor rotamers (%)	0.18	0.17
Ramachandran plot		
Favored (%)	96.15	95.77
Allowed (%)	3.84	4.23
Disallowed (%)	0.01	0.00

Supplementary Table 1 Cryo-EM data collection, refinement and validation statistics
