# Supplementary material for: In situ structures of rotavirus polymerase in action and mechanism of mRNA transcription and release
Source: Nat Commun. 2019 May 17;10:2216. doi: 10.1038/s41467-019-10236-7 (PMC6525196; doi:10.1038/s41467-019-10236-7)
Supplement: Supplementary file 2 — Description of Additional Supplementary Files [file 41467_2019_10236_MOESM2_ESM.pdf]

Description of Additional Supplementary Files

File name: Supplementary Movie 1
Description: Structure of transcribing DLP at near atomic resolution illustrating the quality of the cryoEM sub-particle reconstruction.

File name: Supplementary Movie 2
Description: CryoEM density map (grey) superimposed with atomic model highlighting major and minor grooves in dsRNA.

File name: Supplementary Movie 3
Description: Flying-in view of the ribbon diagram of the RdRp atomic model coloured by domains.

File name: Supplementary Movie 4
Description: Flying-in view of the cap-binding site and template entry channel at both DOS and TES showing RNA changes.

File name: Supplementary Movie 5
Description: Flying-in view of the active site of RdRp in DOS and TES. Note that the priming loop stays retracted in both states.

File name: Supplementary Movie 6
Description: RNA trajectory in TES. The movie begins with atomic models (ribbon) showing the trajectory of the template strand RNA and that of the newly transcribed RNA. Then the cryoEM map (gray) is superimposed with the atomic models to show that RNA is resolved in the template entry, template exit and transcript exit channels to support modeling.

File name: Supplementary Movie 7
Description: Morphing of structures between DOS and TES. RdRp structures are shown first, followed by the CSP-A1 structures, both showing drastic conformational changes needed for the release of the transcript in TES.

File name: Supplementary Movie 8
Description: Ribbon diagrams of the RdRp together with its associated CSP decamer, showing that the extended N-terminal regions of three (at TES) or four (at DOS) CSP-As have extensive interactions with RdRp. The one of the orange CSP and that of the green CSP undergo large-scale translocations between the two states to interact with different regions of RdRp.
